# Three-dimensional cardiac microtissues composed of cardiomyocytes and endothelial cells co-differentiated from human pluripotent stem cells

**DOI:** 10.1242/dev.143438

**Published:** 2017-03-15

**Authors:** Elisa Giacomelli, Milena Bellin, Luca Sala, Berend J. van Meer, Leon G. J. Tertoolen, Valeria V. Orlova, Christine L. Mummery

**Affiliations:** 1 Department of Anatomy and Embryology, Leiden University Medical Center, Leiden 2333ZC, The Netherlands; 2 Department of Applied Stem Cell Technologies, University of Twente, Enschede 7500AE, The Netherlands

**Keywords:** Cardiac microtissue (MT), Human pluripotent stem cell-derived endothelial cells, Human pluripotent stem cell-derived cardiomyocytes, Mesoderm induction, Three-dimensional culture model

## Abstract

Cardiomyocytes and endothelial cells in the heart are in close proximity and in constant dialogue. Endothelium regulates the size of the heart, supplies oxygen to the myocardium and secretes factors that support cardiomyocyte function. Robust and predictive cardiac disease models that faithfully recapitulate native human physiology *in vitro* would therefore ideally incorporate this cardiomyocyte-endothelium crosstalk. Here, we have generated and characterized human cardiac microtissues *in vitro* that integrate both cell types in complex 3D structures. We established conditions for simultaneous differentiation of cardiomyocytes and endothelial cells from human pluripotent stem cells following initial cardiac mesoderm induction. The endothelial cells expressed cardiac markers that were also present in primary cardiac microvasculature, suggesting cardiac endothelium identity. These cell populations were further enriched based on surface markers expression, then recombined allowing development of beating 3D structures termed cardiac microtissues. This *in vitro* model was robustly reproducible in both embryonic and induced pluripotent stem cells. It thus represents an advanced human stem cell-based platform for cardiovascular disease modelling and testing of relevant drugs.

## Abstract (precis)

**Summary:** Co-differentiation of endothelial cells and cardiomyocytes from human pluripotent stem cells provides a cardiac microtissue model with potential applications for disease modelling and drug discovery.

## INTRODUCTION

Differentiation of human pluripotent stem cells (hPSCs) towards the cardiac lineage offers great potential for studying human heart development *in vitro* and for developing complex models of cardiovascular diseases. Furthermore, hPSC-derived cardiomyocytes have been widely used as platform for developing cardiovascular toxicity tests *in vitro* (Abassi et al., 2012; Caspi et al., 2009; Guo et al., 2011; Pointon et al., 2013; Rolletschek, 2004; Zeevi-Levin et al., 2012). However, multiple cell types are required to build physiologically relevant tissues *in vivo* and drug-induced cardiotoxicity can have a multicellular component (Cross et al., 2015). For the heart, this means that crosstalk between diverse cell populations, such as the one between cardiac myocytes and endothelial cells of the myocardial vasculature, needs to be captured in a truly representative model (Tirziu et al., 2010).

In development, both cardiomyocytes and endothelial cells originate from lateral plate mesoderm (Garry and Olson, 2006; Moretti et al., 2006). After they form, they communicate via a variety of paracrine, autocrine and endocrine factors. Cardiac endothelium regulates cardiomyocyte metabolism, survival and contractile functions (Brutsaert, 2003; Narmoneva et al., 2004), as well as the delivery of oxygen and free fatty acids to cardiomyocytes (Aird, 2007). Faithful recapitulation of the cardiac tissue environment not only requires consideration of dynamic factors, such as motion and stretch, and electrical communication, but also paracrine signals derived from myocardial endothelial cells (Ravenscroft et al., 2016).

Under physiological conditions, cells are part of a versatile and dynamic network that cannot be recapitulated entirely in two-dimensional (2D) monolayer culture (Abbott, 2003). In this regard, scaffold-free tissue-engineering approaches offer unique opportunities for developing three-dimensional (3D) models of the heart muscle in a microtissue (MT) structure. In this format, cardiomyocytes can be seeded alone or in combination with other cardiac cell types, allowing cell aggregation and subsequent tissue formation, and mimicking the native physiological state (Fennema et al., 2013).

The ability of endothelial cells to enhance maturity and pharmacological function of both primary and hPSC-derived cardiomyocytes has been shown in several cardiac tissue models derived from hanging drop cultures, hydrogels, cell sheets and patches (Caspi et al., 2007; Masumoto et al., 2016; Narmoneva et al., 2004; Ravenscroft et al., 2016; Stevens et al., 2009; Tulloch et al., 2011). However, the majority of these approaches used primary cells derived from either human- or non-human sources, as well as non-cardiac-specific endothelial cell types. How endothelial cells, specifically those of the heart, affect hPSC-cardiomyocyte maturation has not been investigated in depth.

Here, we developed a method that allows MTs to form from cardiomyocytes derived from both human embryonic stem cells (hESCs) and human induced pluripotent stem cells (hiPSCs) cultured alone (MT-CM) or in combination with human stem cell-derived endothelial cells generated from the same cardiac mesoderm (MT-CMEC). This co-differentiation approach yielded endothelial cells with a cardiac identity. To improve robustness and reproducibility of the system, cell populations were enriched before MT formation and recombined in different ratios. After 7 to 20 days in culture, further evidence of maturity, specifically for MT-CMEC, was shown with increased expression of cardiac genes encoding ion channels and Ca^2+^-handling proteins. In addition, microtissues showed a human dose-response to β-adrenoceptor stimulation, responded to increasing stimulation frequency and displayed negative inotropy after treatment with the Ca^2+^-channel blocker verapamil.

Collectively, our data show the potential of this microtissue model for studying human heart development *in vitro* and for developing complex models of cardiovascular diseases in which either cardiomyocytes or endothelial cells are affected.

## RESULTS AND DISCUSSION

### Human pluripotent stem cells can be simultaneously differentiated into cardiomyocytes and endothelial cells from cardiac mesoderm

**Fig. 1.**
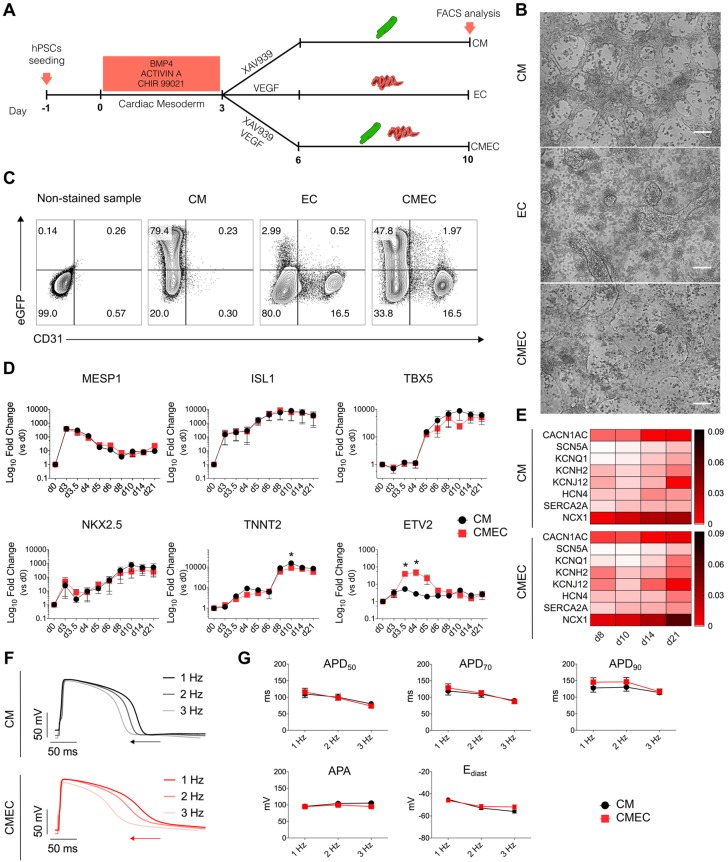
**Simultaneous induction of cardiomyocytes and endothelial cells from cardiac mesoderm.** (A) The differentiation protocol towards cardiomyocyte and endothelial cell fates. Cardiac mesoderm was induced with BMP4, activin A and CHIR 99021 from day 0 to day 3, followed by treatment with XAV939 (CM), VEGF (EC) or XAV939+VEGF (CMEC). (B) Bright-field images of day 10 differentiated *NKX2-5*^eGFP/w^ hESCs under CM, EC and CMEC conditions. Scale bars: 100 μm. (C) Representative FACS plots for CD31 together with eGFP of CM, EC and CMEC populations measured in *NKX2-5*^eGFP/w^ hESCs on day 10 of differentiation. Numbers in the quadrants represent the respective percentage of cells. *n*=4. (D) qRT-PCR analysis at the indicated time points (d=day) for selected cardiac genes under CM (black) and CMEC (red) conditions. Values are normalized to *RPL37A* and relative to undifferentiated NKX2.5^eGFP/w^ hESCs. Two-way ANOVA with Sidak's multiple comparisons test. *P<0.05. *n*=3. Data are mean±s.e.m. (E) Heatmap showing qRT-PCR analysis of key genes encoding ion channels involved in AP shaping and Ca^2+^-handling proteins (linear scale). Values are normalized to *RPL37A* and *TNNT2*, and are relative to undifferentiated NKX2.5^eGFP/w^ hESCs. (F,G) Representative AP traces at 1, 2 and 3 Hz (F), and AP parameter quantification of day 21 *NKX2-5*^eGFP/w^ hESC cardiomyocytes differentiated under CM (black) and CMEC (red) conditions (G). Two-way ANOVA with Sidak's multiple comparisons test. Data are mean±s.e.m. *n*=16-24 from three independent differentiations each.

In order to develop an efficient protocol for the simultaneous differentiation of hPSCs into cardiomyocytes and endothelial cells from cardiac mesoderm, we used the NKX2.5^eGFP/w^ hESC line in which enhanced green fluorescent protein (eGFP) is targeted to the genomic locus of the cardiac transcription factor *NKX2.5* (Elliott et al., 2011). This allows the appearance and enrichment of cardiomyocytes to be monitored using eGFP expression. Cardiac mesoderm was induced in monolayer culture using a combination of bone morphogenetic protein 4 (BMP4, 20 ng/ml), activin A (20 ng/ml) and a small-molecule inhibitor of glycogen synthase kinase-3β (CHIR 99021, 1.5 μM) on days 0-3, followed by inhibition of WNT signalling with XAV939 (5 μM) on days 3-6 (Elliott et al., 2011; van den Berg et al., 2016). On day 3 of differentiation, three distinct conditions were tested: (1) differentiation towards cardiomyocyte cell fate (XAV939 from days 3-6 or CM condition); (2) differentiation towards endothelial cell fate (VEGF from days 3-6 or EC condition); and (3) simultaneous differentiation towards endothelial and cardiomyocyte cell fates (XAV939+VEGF from days 3-6 or CMEC condition) (Fig. 1A). Differentiating cell populations were then refreshed on days 6 and 9 with either growth factor-free (CM) or VEGF supplemented (EC and CMEC) medium. Visual assessment of contracting areas (Fig. 1B; Movies 1 and 2) and fluorescence-activated cell sorting (FACS) (Fig. 1C) on day 10 of differentiation revealed that inhibition of WNT signalling was required to form contracting network-like structures, which were composed of ∼80% and ∼50% eGFP^+^ cardiomyocytes in CM and CMEC conditions, respectively. VEGF was required for endothelial cell specification, resulting in ∼16% eGFP^−^CD31^+^ endothelial cells in EC and CMEC conditions (Fig. 1C). These results demonstrated that inhibition of WNT signalling or VEGF supplementation did not affect endothelial cell or cardiomyocyte formation, respectively. Moreover, VEGF supplementation in the CMEC condition promoted endothelial cell formation at the expense of cardiomyocyte differentiation.

We next performed a time-course experiment to compare the expression of cardiac mesoderm and cardiac-specific genes (*MESP1*, *ISL1*, *TBX5*, *NKX2.5* and *TNNT2*) in CM and CMEC conditions (Fig. 1D). Importantly, expression of these genes started simultaneously in the two groups and followed a similar pattern: *MESP1* peaked on day 3, followed by *ISL1* and *TBX5* induction on days 3 and 5, respectively; *NKX2.5* and *TNNT2* expression was observed from day 8 onwards. As expected, higher expression of *NKX2.5* and *TNNT2* was observed in the CM condition compared with CMEC, confirming the FACS data. *ETV2*, a master regulator of endothelial cell specification, was induced only in the CMEC condition and reached a peak 24 h after VEGF was first added, confirming previous reports that *ETV2* is activated by VEGF (Orlova et al., 2014; Rasmussen et al., 2012) (Fig. 1D).

Expression of key genes encoding ion channels involved in the generation of the cardiac action potential (AP), such as *CACNA1C*, *SCN5A*, *KCNQ1*, *KCNH2*, *KCNJ12* and *HCN4*, as well as genes encoding Ca^2+^-handling proteins (*SERCA2A* and *NCX1*, also known as *ATP2A2* and *SLC8A1*, respectively) started to appear around day 8 and increased over the time until day 21 (Fig. 1E). No significant differences in gene expression were observed between cardiomyocytes derived under CM or CMEC conditions.

In addition, to characterize the electrical phenotype of these cardiomyocytes, we measured the AP of dissociated cells on day 21 using patch-clamp electrophysiology (Fig. 1F,G; Fig. S1). Representative APs elicited at 1, 2 and 3 Hz are shown in Fig. 1F; AP duration (APD), AP amplitude (APA) and diastolic membrane potential (E_diast_) did not differ between the two groups (Fig. 1G; Fig. S1), suggesting that electrophysiological properties of cardiomyocytes generated in CM and CMEC conditions were comparable.

Using the hESC pre-cardiac MESP1 reporter line (Den Hartogh et al., 2014), we have previously demonstrated that *MESP1*^mCherry+^ progenitors can be differentiated into cardiomyocytes, endothelial cells and smooth muscle cells. Time-course analysis confirmed induction of early cardiovascular progenitor markers (*MESP1*, *TBX5* and *ISL1*) in our present differentiation protocol. Therefore, VEGF supplementation from day 3 causes endothelial cells induced from MESP1^+^ISL1^+^ cardiovascular progenitors to be directed specifically to a cardiac endothelial cell fate. We further demonstrated that VEGF supplementation did not affect cardiomyocyte specification and function, as shown by cardiac ion-channel expression and electrophysiological properties. Collectively, our data demonstrated that both cardiomyocytes and endothelial cells can be differentiated simultaneously from early cardiac mesoderm.

### Characterization of CD34^+^ hPSC-derived endothelial cells isolated from cardiac mesoderm

To develop a reliable 3D model of cardiac tissue, we aimed to isolate cardiac endothelial cells derived as above from the heterogeneous differentiated hPSC cultures and mix these with cardiomyocytes in defined ratios. Because CD34, together with VE-cadherin (VEC), is one of the earliest markers of endothelial cell progenitors (Choi et al., 2012; Lian et al., 2014; Orlova et al., 2014), we first performed a time-course experiment to identify optimal differentiation conditions, timing and cell-seeding density for the induction of CD34^+^ endothelial cells (Fig. S2A). Notably, the highest percentage of endothelial cells was observed on day 6 of differentiation in the CMEC condition, by seeding 12.5×10^3^ cells per cm^2^. Based on these findings, we isolated CMEC-derived endothelial cells on day 6, using a simple procedure of immunomagnetic selection with anti-CD34 antibody–coupled magnetic beads (Lian et al., 2014). To test whether the same protocol could be applied to hiPSC, we used both NKX2.5^eGFP/w^ hESC and wild-type hiPSC described previously (Zhang et al., 2014).

**Fig. 2.**
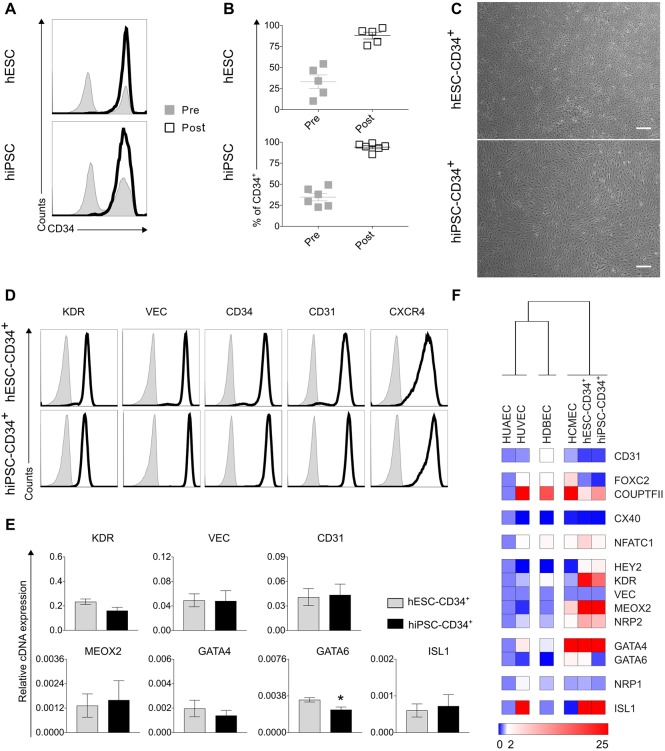
**Isolation and characterization of endothelial cells.** (A,B) FACS histograms from representative experiments (A) and averaged percentages from multiple experiments (B) of CD34^+^ cells in the pre-isolation (grey) and post-isolation (black) fractions showing the efficiency of the isolation strategy. Experiments were performed on cells derived from *NKX2-5*^eGFP/w^ hESCs (upper panels, *n*=5) and hiPSCs (lower panels, *n*=6). (C) Representative bright-field images of the morphological appearance of CMEC-derived CD34^+^ cells from *NKX2-5*^eGFP/w^ hESCs (upper panel) and hiPSCs (lower panel) after isolation and re-plating. Scale bars: 200 μm. (D) FACS measurement (histograms) for key endothelial cell-surface markers of CD34^+^ cells 4 days after isolation and re-plating. Specific antibody-labelled cells are shown in black (*NKX2-5*^eGFP/w^ hESC line, upper panels; hiPSC line, lower panels) (*n*=3). (E) qRT-PCR analysis for key endothelial genes (upper panels) and for cardiac-specific genes (lower panels) in CMEC-derived CD34^+^ cells from *NKX2-5*^eGFP/w^ hESCs (grey) and hiPSCs (black). Mann–Whitney test. *P=0.0286. *n*>3. Data are mean±s.e.m. Values are normalized to *RPL37A*. (F) qRT-PCR analysis (heatmap) and hierarchical clustering showing a panel of endothelial and cardiac genes of interest in HUAECs, HUVECs, HDBECs and HCMECs together with CMEC-derived CD34^+^ cells from NKX2.5^eGFP/w^ hESCs and hiPSCs (*n*=3). Values are normalized to *RPL37A* and *VEC*, and are relative to HUAECs.

To determine the extent of CD34^+^ endothelial cell enrichment after isolation, we performed FACS analysis on the cell suspensions before and after purification (Fig. 2A,B). Significant enrichment with >95% CD34^+^ endothelial cell purity was achieved in the post-isolation fraction. When subsequently plated, the isolated CD34^+^ cells were highly proliferative, reached confluence within 3 or 4 days and displayed typical endothelial morphology (Fig. 2C). Moreover, FACS analysis revealed expression of key endothelial cell-surface markers, such as KDR, VEC, CD34 and CD31, in both hESC- and hiPSC-derived CD34^+^ endothelial cells (Fig. 2D). Notably, these cells were also positive for the arterial marker CXCR4. At this stage, endothelial cells were suitable for either MT formation or, alternatively, cryopreservation for later use (Fig. S2B).

To characterize endothelial cells isolated on day 6, we determined the expression of typical endothelial markers such as *KDR*, *VEC* and *CD31*, as well as cardiac-specific markers, such as *MEOX2*, *GATA4*, *GATA6* and *ISL1*, by quantitative RT-PCR (qRT-PCR) (Fig. 2E). These day 6 CD34^+^ endothelial cells derived from both hESC and hiPSC exhibited comparable marker expression. Moreover, when compared with primary endothelial cells such as human umbilical artery endothelial cells (HUAECs), human umbilical vein endothelial cells (HUVECs), human dermal blood endothelial cells (HDBECs) and human cardiac microvascular endothelial cells (HCMECs), day 6 CD34^+^ endothelial cells clustered with HCMEC and showed similar expression of the cardiac-specific marker *GATA4* (Fig. 2F)*.* This is consistent with previously reported data demonstrating that *GATA4* is crucial for heart formation during embryonic development and strongly implicated in congenital heart diseases (Butler et al., 2010; Furtado et al., 2016; Garg et al., 2003), and suggests a cardiac endothelium identity for the CMEC-derived endothelial cells.

Endothelial cells can differentiate from different types of mesoderm. In the heart, they originate from both endocardial and second heart-field progenitors (Misfeldt et al., 2009; Sahara et al., 2015). Importantly, in the developing postnatal mouse heart, endocardium contributes to more than 70% of coronary endothelium (Espinosa-Medina et al., 2014; Tian et al., 2015). Although very little is known about the developmental and the genetic signature of human endocardial (endothelial) cells, lineage tracing has demonstrated that these cells originate from both multipotent cardiac progenitors and early cardiac mesoderm (Misfeldt et al., 2009). We therefore investigated whether we could differentiate endothelial cells from a common cardiac mesoderm. Interestingly, we found that day 6 endothelial progenitors exhibited a similar genetic signature to human primary cardiac endothelial cells (Fig. 2F). Most strikingly, increased expression of *MEOX2*, *GATA4*, *GATA6* and *ISL1* was observed compared with primary non-cardiac endothelial cells. Future genome-wide transcriptional studies will be required for in depth characterization of the cardiac/endocardial origin of these endothelial cells. Other stimuli, such as the upregulation of fatty acid transporters, could help to determine whether co-culture with cardiomyocytes can shift their cardiac endothelial cell profile closer to *bona fide* cardiac endothelial cells (Coppiello et al., 2015; Hagberg et al., 2013; Jang et al., 2016).

### hPSC-VCAM1-enriched cardiomyocytes display typical sarcomeric structures and cardiac electrophysiological properties

**Fig. 3.**
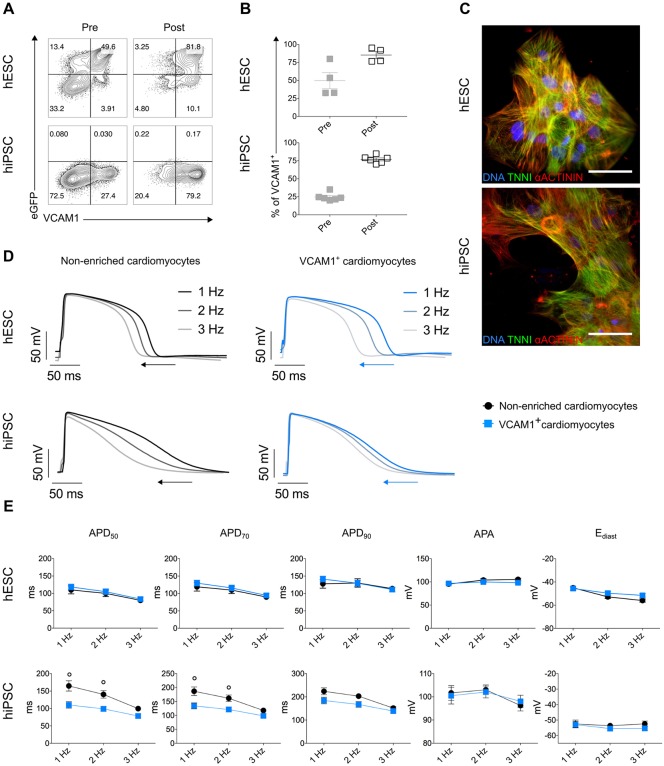
**Isolation and characterization of cardiomyocytes.** (A,B) FACS plots from representative experiments analysing VCAM1 and eGFP (A), and average percentages from multiple experiments of VCAM1^+^ cells (B) in the pre-isolation (grey) and post-isolation (black) fractions showing the efficiency of the isolation strategy. Experiments were performed on cells differentiated from *NKX2-5*^eGFP/w^ hESCs (upper panels, *n*=4) and hiPSCs (lower panels, *n*=6). (C) Immunofluorescence images of cardiac sarcomeric proteins TNNI (green) and α-actinin (red) in VCAM1^+^ cardiomyocytes generated from NKX2.5^eGFP/w^ hESCs (upper panel) and hiPSCs (lower panel). Nuclei are stained in blue with DAPI. Scale bars: 50 μm. (D,E) Representative APs at 1, 2 and 3 Hz (D), and AP parameter quantification of non-enriched (black) and VCAM1^+^ (blue) cardiomyocytes differentiated from *NKX2-5*^eGFP/w^ hESCs (upper panels, *n*=16 and 16) and hiPSCs (lower panels, *n*=15 and 18) from three independent differentiations each (E). Two-way ANOVA with Sidak's multiple comparisons test. *P*<0.05 versus VCAM1^+^ cardiomyocytes. Data are mean±s.e.m.

In order to obtain a defined cardiomyocyte population, we used VCAM1, previously described as a cardiomyocyte surface marker, to isolate VCAM1^+^ cells from differentiated cultures (Skelton et al., 2014; Uosaki et al., 2011; Wang et al., 2014). As with the endothelial purification strategy, we first performed time-course analysis using NKX2.5^eGFP/w^ hESC to identify optimal differentiation conditions, timing and cell-seeding density for the differentiation of VCAM1^+^ cardiomyocytes in CM and CMEC conditions (Fig. S3A). Cells from both conditions showed initial expression of VCAM1 at day 10, reaching a maximum on day 14, in agreement with previous studies on hESC differentiation (Skelton et al., 2014). The CM condition resulted in higher numbers of eGFP^+^VCAM1^+^ cells compared with the CMEC condition. Moreover, the highest percentage of cardiomyocytes was observed by seeding 25×10^3^ cells per cm^2^. Using these conditions for both NKX2.5^eGFP/w^ hESC and the wild-type hiPSC, VCAM1^+^ cells were enriched by immunomagnetic selection with anti-VCAM1-PE-labelled antibody-coupled magnetic beads. FACS analysis on cell suspensions before and after isolation revealed ∼80% enrichment of VCAM1^+^ cells (Fig. 3A,B). Importantly, after bead sorting, VCAM1^+^ cells re-plated in culture re-formed spontaneously contracting networks within 3-4 days (Fig. S3B; Movie 3), accompanied by NKX2.5-eGFP expression in the hESC line (Fig. S3C). Furthermore, VCAM1^+^ cells displayed characteristic sarcomeric structures that stained positively for troponin I (TNNI) and α-actinin (Fig. 3C).

Electrophysiological properties of VCAM1^+^ cardiomyocytes were next compared with non-enriched cardiomyocytes (Fig. 3D,E; Fig. S3D). Current clamp measurements revealed no significant differences between the two groups in the NKX2.5^eGFP/w^ hESC line (Fig. 3D,E; Fig. S3D). In the hiPSC line, although VCAM1^+^ cells displayed shorter APD_50_ and APD_70_ at 1 and 2 Hz, they did not show significant differences in APD_90_, APA and E_diast_ when compared with the non-enriched population (Fig. 3D,E; Fig. S3D).

Enrichment of the cardiac population from differentiated cultures based on VCAM1 expression has been previously described (Elliott et al., 2011; Schwach and Passier, 2016; Skelton et al., 2014; Uosaki et al., 2011; Wang et al., 2014), but the electrical phenotype of purified cardiomyocytes had not been assessed to date. Here, we optimised a protocol based on anti-PE magnetic nanoparticles, which allowed isolation of VCAM1^+^ cardiomyocytes with high viability, and supported maintenance of their typical sarcomeric structure and electrophysiological properties ready for downstream applications.

### hPSC-derived endothelial cells and cardiomyocytes form 3D contracting cardiac microtissues

**Fig. 4.**
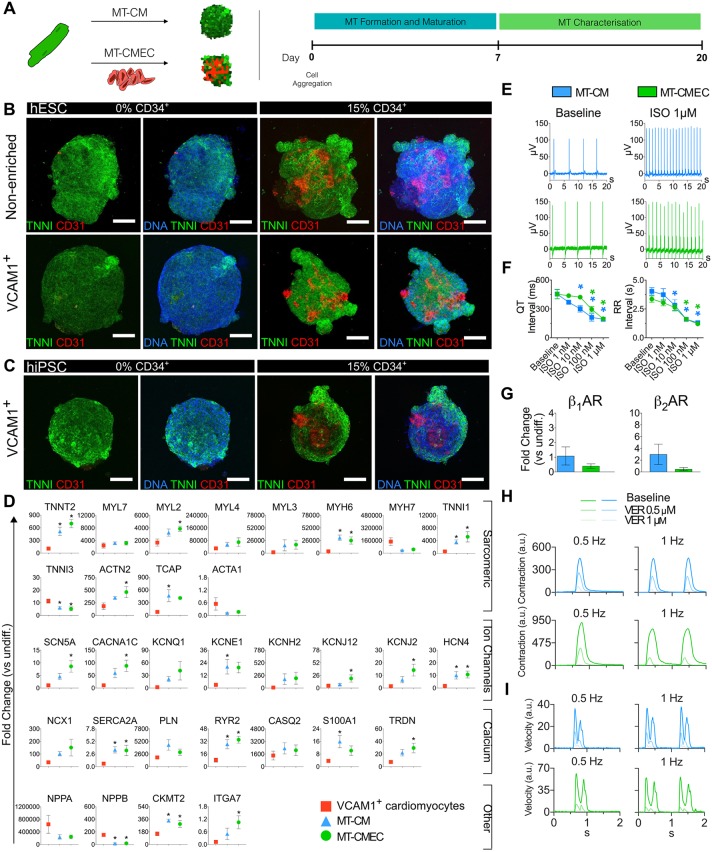
**Generation and characterization of 3D cardiac microtissues.** (A) The protocol to generate cardiac MTs from cardiomyocytes cultured alone (MT-CM) or in combination with enriched CD34^+^ endothelial cells (MT-CMEC). MTs from *NKX2-5*^eGFP/w^ hESCs were generated from non-enriched or enriched VCAM1^+^ cardiomyocytes, whereas MTs from hiPSCs were generated from enriched VCAM1^+^ cardiomyocytes only. MT characterization was performed between days 7 and 20 by immunofluorescence, qRT-PCR, MEAs and contraction analyses. (B) Immunofluorescence analysis of sarcomeric cardiac TNNI (green) and endothelial cell surface marker CD31 (red) of day 7 cardiac MTs from non-enriched (upper panels) and VCAM1-enriched (lower panels) cardiomyocytes from *NKX2-5*^eGFP/w^ hESCs. Percentages of CD34^+^ cells are shown at the top. Scale bars: 100 μm. (C) Immunofluorescence analyses of TNNI (green) and CD31 (red) of day 7 MTs generated from hiPSC-VCAM1^+^ cardiomyocytes. Percentages of CD34^+^ cells are shown at the top. Scale bars: 100 μm. (D) qRT-PCR analysis for key sarcomeric genes, ion channels involved in AP shaping and Ca^2+^ regulatory genes, as well as other cardiac genes of interest in day 7 hiPSC-MTs and in day 21 age-matched VCAM1^+^ cardiomyocytes from hiPSCs. All values are normalized to *RPL37A* and relative to undifferentiated hiPSCs. Data are mean±s.e.m., *n*>4. One-way ANOVA with Tukey's multiple comparisons test. *P<0.05 versus VCAM1^+^ cardiomyocytes. (E) FP representative traces measured using MEAs under baseline conditions (left panels) and upon addition of 1 μM isoprenaline (ISO) (right panels) in MT-CM (upper panels, blue) and MT-CMEC (lower panels, green) from hiPSCs. (F) QT and RR intervals measured using MEAs under baseline conditions and after increasing concentrations of ISO in MT-CM (blue) and MT-CMEC (red) from hiPSCs. One-way ANOVA. *P<0.05 versus baseline. Colour code of the asterisks indicates the experimental group. Data are mean±s.e.m., *n*=9. (G) qRT-PCR analysis of β-adrenoreceptors (β_1_ AR, left panel; β_2_ AR, right panel) in day 7 MT-CM and MT-CMEC from hiPSCs. Values are normalized to *RPL37A* and are relative to undifferentiated hiPSCs. Mann–Whitney test. Data are mean±s.e.m., *n*=3. (H,I) Representative traces of contraction (H) and contraction velocity (I) in MT-CM (blue) and MT-CMEC (green) generated from hiPSCs and paced at 0.5 (left panels) and 1 Hz (right panels). Results are shown under baseline conditions and after superfusion of 500 nM and 1 µM verapamil (VER).

To optimize conditions for generating cardiac MTs, we first used either non-enriched or VCAM1-enriched hESC-cardiomyocytes either alone (MT-CM) or in combination with hESC-derived CD34^+^ endothelial cells (MT-CMEC). Spheroid MTs were formed in V-bottomed 96-well microplates and were refreshed every 3 days with either growth factor-free (MT-CM) or VEGF-supplemented (MT-CMEC) medium. MTs were characterized between days 7 and 20 after initial aggregation (Fig. 4A).

To determine the morphology and the cellular architecture of both MT-CM and MT-CMEC, we performed immunostaining for cardiomyocyte- and endothelial-specific cell markers (using TNNI and CD31 antibodies, respectively). In addition, to define optimal endothelium/myocyte ratios within the MTs, different percentages of cardiomyocytes and CD34^+^ cells were combined (Fig. 4B; Fig. S4A). Interestingly, immunohistochemistry revealed that MTs composed of 15% endothelial cells and 85% cardiomyocytes resulted in a better endothelial cell organization and distribution within the MT compared with MTs containing 40% endothelial cells (Fig. S4A). After optimizing conditions for MT formation using hESCs, we used the same protocol for wild-type hiPSCs. Immunofluorescence analysis confirmed TNNI and CD31 expression in 3D MTs generated from VCAM1^+^ cardiomyocytes in combination with 15% CD34^+^ endothelial cells (Fig. 4C). Importantly, VCAM1^+^ bead-sorted cardiomyocytes maintained their ability to form 3D aggregates alone or in combination with endothelial cells.

Next, to investigate whether gene expression was changed by 3D organization and/or the presence of endothelial cells, we compared expression of a broad panel of genes in MT-CM and MT-CMEC with 2D monolayer VCAM1^+^ cardiomyocytes by qRT-PCR. Specifically, we quantified expression of genes involved in sarcomere assembly (*MYL2*, *MYL7*, *MYL4*, *MYL3*, *MYH6*, *MYH7*, *TNNI1*, *TNNI3*, *ACTN2*, *TCAP* and *ACTA1*), in cardiac AP (*SCN5A*, *CACNA1C*, *KCNQ1*, *KCNE1*, *KCNH2*, *KCNJ12*, *KCNJ2* and *HCN4*) and in Ca^2+^ handling (*NCX1*, *SERCA2A*, *PLN*, *RYR2*, *CASQ2*, *S100A1* and *TRDN*). In addition, expression of fetal cardiomyocyte-enriched genes (*NPPA* and *NPPB*), as well as the sarcomeric mitochondrial gene *CKMT2* (Babiarz et al., 2012; Chun et al., 2015; Payne and Strauss, 1994) and the α7 integrin subunit (*ITGA7*), were also quantified (Fig. 4D; Fig. S4B).

After 7 days in culture, MT-CMEC showed gene expression changes associated with progression of heart development and fetal-to-postnatal transition: upregulation of the sarcomeric structural genes *TNNT2*, *MYL2*, *ACTN2* and *TCAP*; upregulation of the ion-channel genes *SCN5A*, *CACNA1C*, *KCNJ12* and *KCNJ2*; upregulation of the Ca^2+^-handling genes *SERCA2A*,* RYR2* and *TRDN*; upregulation of *CKMT2* and *ITGA*; and downregulation of the fetal-enriched gene *NPPB*. Conversely, changes in other genes not associated with advanced maturation were also observed: upregulation of the fetal sarcomeric structural genes *MYH6* and *TNNI1*; upregulation of *HCN4*; and downregulation of the adult sarcomeric structural gene *TNNI3*. A similar trend for the majority of genes was observed in MTs generated from hESCs, although not all changes were consistent with hiPSC-MTs (Fig. S4B).

Furthermore, to investigate whether maturation increased with time in culture, we generated MTs from both hESCs and hiPSCs, and cultured them for 20 days (Fig. S5). Gene expression analysis at this time point showed upregulation of cardiac ion-channel genes (*SCN5A*, *CACNA1C*, *KCNQ1* and *KCNE1*), upregulation of the Ca^2+^-handling genes *CASQ2* and *TRDN* as well as downregulation of the foetal cardiomyocyte-enriched genes *MYH6* and *TNNI1* (Fig. S5A,B). In addition, day 20 MTs from both hESCs and hiPSCs displayed increased *MYL2/MYL7* and *MYH7/MYH6* ratios when compared with day 7 MTs (Fig. S5C,D).

Taken together, our results suggested that 3D culture organization, inclusion of endothelial cells and prolonged time in culture induced crucial changes in the gene expression of cardiomyocytes that were associated with maturation in our *in vitro* cardiac microtissue system.

Next, to investigate the electrical phenotype of MT-CM and MT-CMEC, we measured QT and RR intervals using multielectrode array (MEA) at baseline and following addition of increasing concentrations of the β-adrenoreceptor agonist isoprenaline (ISO) (Fig. 4E,F; Fig. S6). Representative field potential (FP) recordings at baseline and after addition of 1 μM ISO are shown in Fig. 4E. Interestingly, QT and RR intervals did not differ between the MT-CM and MT-CMEC groups (Fig. 4F), and the dependence of QT interval duration from the RR interval was uniform in the two groups (Fig. S6), suggesting that the baseline electrical properties of the MT were conserved with or without endothelial cells. However, in MT-CM, 10 nM ISO significantly shortened QT and RR intervals compared with baseline values, whereas MT-CMEC required a higher concentration of ISO (100 nM) to undergo significant shortening. This might be due to the lower expression of β_1_ and β_2_ adrenoreceptor genes (*ADRB1* and *ADRB2*, respectively) in MT-CMEC compared with MT-CM, which might be linked to the different cell composition (Fig. 4G; Fig. S7A).

Finally, qualitative analysis of MT contraction was performed in paced hiPSC-MTs and in hESC-MTs at 0.5 and 1 Hz (Fig. 4H,I; Fig. S7B,C), under baseline conditions and after treatment with the Ca^2+^-channel blocker verapamil (VER, 500 nM and 1 µM). Notably, VER decreased the contraction amplitude and velocity, as expected from the block of the L-type Ca^2+^ channels and as observed earlier in MTs with primary endothelial cells (Ravenscroft et al., 2016).

Taken together, we conclude that the *in vitro* cardiac microtissue that we have developed represents a robust system suitable for medium- to large-scale production and a valid tool for studying cardiomyocyte maturation, disease modelling and drug screening. Importantly, additional studies are required to assess full cardiomyocyte maturation, including sarcomeric organization, mitochondria content, and replication of the physiological and pharmacological responses that are typical of the native human heart tissue. Compared with existing systems (Table S3), ours has both limitations and advantages. For example, compared with engineered heart tissues (EHTs), microtissues required a substantially smaller number of cells and therefore are more amenable to large-scale production. However, EHTs display cell and sarcomeric alignment, and allow the measurement of contractile force; EHTs have been able to recapitulate the positive and negative inotropic effects of molecules and drugs in the heart (Mannhardt et al., 2016). Micro-heart muscles combine the advantages of scalability, cell alignment and force measurement (Huebsch et al., 2016).

Undoubtedly, increasing the complexity of our microtissue format by inclusion of other cardiac cell types and complex 3D architectures, or even by implementation of fluid flow for the endothelial cells has to be explored to further improve the system. In addition, the fact that cardiomyocytes and endothelial cells possess different metabolic states also needs to be taken into account: on the one hand, embryonic or immature cardiomyocytes are highly dependent on glycolysis, whereas maturation is associated with the switch towards fatty acid oxidation; on the other hand, endothelial cells are highly dependent on glycolysis for active angiogenesis (Schoors et al., 2014). Therefore, the correct balance between glucose and free fatty acid supplementation will be essential to promote further maturation in our system. Furthermore, as endothelial cells serve as a semi-permeable barrier for the delivery of nutrients to the cardiomyocytes, a separation of either the fluid or the nutrient compartment might be essential to prevent direct contact with cardiomyocytes. Finally, it will be interesting to investigate whether maturation of endothelial cells is also improved in the MT system described here.

## MATERIALS AND METHODS

### hPSC lines culture and differentiation into endothelial cells and cardiomyocytes

Previously described NKX2-5^eGFP/w^ hESCs and wild-type hiPSCs (Elliott et al., 2011; Zhang et al., 2014) were cultured in E8 medium (Life Technologies). Cardiac and endothelial differentiations were induced in a monolayer: CM condition as previously described (van den Berg et al., 2016; Elliott et al., 2011); details of EC and CMEC conditions are provided above and in the supplementary Materials and Methods.

### FACS analysis

Staining was carried out with the following antibodies: anti-VCAM1-PE, anti-CD34-APC, anti-KDR-PE, anti-VEC-PECY7, anti-CD31-APC and anti-CXCR4-PE. Further details are provided in the supplementary Materials and Methods. Antibodies are listed in Table S2.

### Isolation of CD34^+^ endothelial cells and VCAM1^+^ cardiomyocytes

CD34^+^ cells were isolated using a Human Cord Blood CD34 Positive Selection kit II (StemCell Technologies) whereas VCAM1^+^ cells were isolated using a Human PE Selection kit (StemCell Technologies) following the manufacturer's instructions (see also supplementary Materials and Methods).

### Generation and cultivation of cardiac microtissues

CD34^+^ endothelial cells and enriched or non-enriched VCAM1^+^ cardiomyocytes were prepared prior to microtissue formation and combined for self-aggregation as described in the supplementary Materials and Methods.

### Immunofluorescence analysis

Immunostaining was carried out with the following primary antibodies: TNNI and α-actinin for VCAM1^+^ cardiomyocytes; and CD31 and TNNI for MTs. Primary antibodies were detected using Cy3- and Alexa Fluor 488-conjugated secondary antibodies. Further details are provided in the supplementary Materials and Methods.

### Patch-clamp and multielectrode array electrophysiology

Electrical signals for patch-clamp single cell analysis were recorded with an Axopatch 200B Amplifier (Molecular Devices) and digitized with a Digidata 1440A (Molecular Devices), and MEA experiments were performed using a 64-electrode USB-MEA system (Multichannel Systems) as previously described (Sala et al., 2016). Details are provided in the supplementary Materials and Methods.

### Contraction analysis

Movies of paced MTs were acquired with a ThorLabs DCC3240M camera and analysed with a custom-made algorithm. Details are provided in the supplementary Materials and Methods.

### Drugs

Isoprenaline (Sigma-Aldrich) was dissolved in MilliQ water and verapamil (Sigma-Aldrich) was dissolved in 100% ethanol following the manufacturer's instructions. Stock solutions were freshly prepared before experiments.

### Gene expression analysis

For RT-qPCR, RNA was purified using the RNeasy Mini Kit (Qiagen) and reverse transcribed using the iScript-cDNA Synthesis kit (Bio-Rad). Gene expression was assessed using a Bio-Rad CFX384 real-time system and data were analysed using the ΔΔCt method. Further details are provided in the supplementary Materials and Methods. Primer sequences can be found in Table S1.

### Bright-field images and movies

Bright-field images and movies were acquired with a Nikon DS-2MBW camera connected to a Nikon Eclipse Ti-S microscope controlled by the Nikon NIS-Element BR software. Lens magnification was 4× or 10× with a PhL or Ph1 contrast filter.

### Statistics

Ordinary one-way, two-way ANOVA and Mann–Whitney tests for paired or unpaired measurements were applied for differences in means between groups/conditions. Detailed statistics are indicated in each figure legend. Data are expressed as mean±s.e.m. Statistical significance was defined as *P*<0.05. Further details are provided in the supplementary Materials and Methods.

## References

[DEV143438C1] Abassi Y. A., Xi B., Li N., Ouyang W., Seiler A., Watzele M., Kettenhofen R., Bohlen H., Ehlich A., Kolossov E. et al. (2012). Dynamic monitoring of beating periodicity of stem cell-derived cardiomyocytes as a predictive tool for preclinical safety assessment. *Br. J. Pharmacol.* 165, 1424-1441. 10.1111/j.1476-5381.2011.01623.x 21838757 PMC3372727

[DEV143438C2] Abbott A. (2003). Cell culture: biology's new dimension. *Nature* 424, 870-872. 12931155 10.1038/424870a

[DEV143438C3] Aird W. C. (2007). Phenotypic heterogeneity of the endothelium: II. Representative vascular beds. *Circ. Res.* 100, 174-190. 10.1161/01.RES.0000255690.03436.ae 17272819

[DEV143438C4] Babiarz J. E., Ravon M., Sridhar S., Ravindran P., Swanson B., Bitter H., Weiser T., Chiao E., Certa U. and Kolaja K. L. (2012). Determination of the human cardiomyocyte mRNA and miRNA differentiation network by fine-scale profiling. *Stem Cells Dev.* 21, 1956-1965. 10.1089/scd.2011.0357 22050602 PMC4048009

[DEV143438C5] Brutsaert D. L. (2003). Cardiac endothelial-myocardial signaling: its role in cardiac growth, contractile performance, and rhythmicity. *Physiol. Rev.* 83, 59-115. 10.1152/physrev.00017.2002 12506127

[DEV143438C6] Butler T. L., Esposito G., Blue G. M., Cole A. D., Costa M. W., Waddell L. B., Walizada G., Sholler G. F., Kirk E. P., Feneley M. et al. (2010). GATA4 mutations in 357 unrelated patients with congenital heart malformation. *Genet. Test. Mol. Biomarkers* 14, 797-802. 10.1089/gtmb.2010.0028 20874241

[DEV143438C7] Caspi O., Lesman A., Basevitch Y., Gepstein A., Arbel G., Habib I. H. M., Gepstein L. and Levenberg S. (2007). Tissue engineering of vascularized cardiac muscle from human embryonic stem cells. *Circ. Res.* 100, 263-272. 10.1161/01.RES.0000257776.05673.ff 17218605

[DEV143438C8] Caspi O., Itzhaki I., Kehat I., Gepstein A., Arbel G., Huber I., Satin J. and Gepstein L. (2009). In vitro electrophysiological drug testing using human embryonic stem cell derived cardiomyocytes. *Stem Cells Dev.* 18, 161-172. 10.1089/scd.2007.0280 18510453

[DEV143438C9] Choi K.-D., Vodyanik M. A., Togarrati P. P., Suknuntha K., Kumar A., Samarjeet F., Probasco M. D., Tian S., Stewart R., Thomson J. A. et al. (2012). Identification of the hemogenic endothelial progenitor and its direct precursor in human pluripotent stem cell differentiation cultures. *Cell Reports* 2, 553-567. 10.1016/j.celrep.2012.08.002 22981233 PMC3462245

[DEV143438C10] Chun Y. W., Balikov D. A., Feaster T. K., Williams C. H., Sheng C. C., Lee J.-B., Boire T. C., Neely M. D., Bellan L. M., Ess K. C. et al. (2015). Combinatorial polymer matrices enhance in vitro maturation of human induced pluripotent stem cell-derived cardiomyocytes. *Biomaterials* 67, 52-64. 10.1016/j.biomaterials.2015.07.004 26204225 PMC4550551

[DEV143438C11] Coppiello G., Collantes M., Sirerol-Piquer M. S., Vandenwijngaert S., Schoors S., Swinnen M., Vandersmissen I., Herijgers P., Topal B., van Loon J. et al. (2015). Meox2/Tcf15 heterodimers program the heart capillary endothelium for cardiac fatty acid uptake. *Circulation* 131, 815-826. 10.1161/CIRCULATIONAHA.114.013721 25561514

[DEV143438C12] Cross M. J., Berridge B. R., Clements P. J. M., Cove-Smith L., Force T. L., Hoffmann P., Holbrook M., Lyon A. R., Mellor H. R., Norris A. A. et al. (2015). Physiological, pharmacological and toxicological considerations of drug-induced structural cardiac injury. *Br. J. Pharmacol.* 172, 957-974. 10.1111/bph.12979 25302413 PMC4314188

[DEV143438C13] Den Hartogh S. C., Schreurs C., Monshouwer-Kloots J. J., Davis R. P., Elliott D. A., Mummery C. L. and Passier R. (2014). Dual reporter MESP1 mCherry/w-NKX2-5 eGFP/whESCs enable studying early human cardiac differentiation. *Stem Cells* 33, 56-67. 10.1002/stem.1842 25187301

[DEV143438C14] Elliott D. A., Braam S. R., Koutsis K., Ng E. S., Jenny R., Lagerqvist E. L., Biben C., Hatzistavrou T., Hirst C. E., Yu Q. C. et al. (2011). NKX2-5eGFP/w hESCs for isolation of human cardiac progenitors and cardiomyocytes. *Nat. Meth.* 8, 1037-1040. 10.1038/nmeth.1740 22020065

[DEV143438C15] Espinosa-Medina I., Outin E., Picard C. A., Chettouh Z., Dymecki S., Consalez G. G., Coppola E. and Brunet J.-F. (2014). Parasympathetic ganglia derive from Schwann cell precursors. *Science* 345, 87-90. 10.1126/science.1253286 24925912

[DEV143438C16] Fennema E., Rivron N., Rouwkema J., van Blitterswijk C. and de Boer J. (2013). Spheroid culture as a tool for creating 3D complex tissues. *Trends Biotechnol.*. 31, 108-115. 10.1016/j.tibtech.2012.12.003 23336996

[DEV143438C17] Furtado M. B., Nim H. T., Boyd S. E. and Rosenthal N. A. (2016). View from the heart: cardiac fibroblasts in development, scarring and regeneration. *Development* 143, 387-397. 10.1242/dev.120576 26839342

[DEV143438C18] Garg V., Kathiriya I. S., Barnes R., Schluterman M. K., King I. N., Butler C. A., Rothrock C. R., Eapen R. S., Hirayama-Yamada K., Joo K. et al. (2003). GATA4 mutations cause human congenital heart defects and reveal an interaction with TBX5. *Nature* 424, 443-447. 10.1038/nature01827 12845333

[DEV143438C19] Garry D. J. and Olson E. N. (2006). A common progenitor at the heart of development. *Cell* 127, 1101-1104. 10.1016/j.cell.2006.11.031 17174889

[DEV143438C20] Guo L., Abrams R. M. C., Babiarz J. E., Cohen J. D., Kameoka S., Sanders M. J., Chiao E. and Kolaja K. L. (2011). Estimating the risk of drug-induced proarrhythmia using human induced pluripotent stem cell-derived cardiomyocytes. *Toxicol. Sci.* 123, 281-289. 10.1093/toxsci/kfr158 21693436

[DEV143438C21] Hagberg C. E., Mehlem A., Falkevall A., Muhl L., Fam B. C., Ortsäter H., Scotney P., Nyqvist D., Samén E., Lu L. et al. (2013). Targeting VEGF-B as a novel treatment for insulin resistance and type 2 diabetes. *Nature* 490, 426-430. 10.1038/nature11464 23023133

[DEV143438C22] Huebsch N., Loskill P., Deveshwar N., Spencer C. I., Judge L. M., Mandegar M. A., Fox C. B., Mohamed T. M. A., Ma Z., Mathur A. et al. (2016). Miniaturized iPS-cell-derived cardiac muscles for physiologically relevant drug response analyses. *Sci. Rep.* 6, 24726 10.1038/srep24726 27095412 PMC4837370

[DEV143438C23] Jang C., Oh S. F., Wada S., Rowe G. C., Liu L., Chan M. C., Rhee J., Hoshino A., Kim B., Ibrahim A. et al. (2016). A branched-chain amino acid metabolite drives vascular fatty acid transport and causes insulin resistance. *Nat. Med.* 22, 421-426. 10.1038/nm.4057 26950361 PMC4949205

[DEV143438C24] Lian X., Bao X., Al-Ahmad A., Liu J., Wu Y., Dong W., Dunn K. K., Shusta E. V. and Palecek S. P. (2014). Efficient differentiation of human pluripotent stem cells to endothelial progenitors via small-molecule activation of WNT signaling. *Stem Cell Rep.* 3, 804-816. 10.1016/j.stemcr.2014.09.005 PMC4235141 25418725

[DEV143438C26] Mannhardt I., Breckwoldt K., Letuffe-Brenière D., Schaaf S., Schulz H., Neuber C., Benzin A., Werner T., Eder A., Schulze T. et al. (2016). Human engineered heart tissue: analysis of contractile force. *Stem Cell Rep.s* 7, 29-42. 10.1016/j.stemcr.2016.04.011 PMC4944531 27211213

[DEV143438C27] Masumoto H., Nakane T., Tinney J. P., Yuan F., Ye F., Kowalski W. J., Minakata K., Sakata R., Yamashita J. K. and Keller B. B. (2016). The myocardial regenerative potential of three-dimensional engineered cardiac tissues composed of multiple human iPS cell-derived cardiovascular cell lineages. *Sci. Rep.* 6, 29933 10.1038/srep29933 27435115 PMC4951692

[DEV143438C28] Misfeldt A. M., Boyle S. C., Tompkins K. L., Bautch V. L., Labosky P. A. and Baldwin H. S. (2009). Endocardial cells are a distinct endothelial lineage derived from Flk1+ multipotent cardiovascular progenitors. *Dev. Biol.* 333, 78-89. 10.1016/j.ydbio.2009.06.033 19576203

[DEV143438C29] Moretti A., Caron L., Nakano A., Lam J. T., Bernshausen A., Chen Y., Qyang Y., Bu L., Sasaki M., Martin-Puig S. et al. (2006). Multipotent embryonic isl1+ progenitor cells lead to cardiac, smooth muscle, and endothelial cell diversification. *Cell* 127, 1151-1165. 10.1016/j.cell.2006.10.029 17123592

[DEV143438C30] Narmoneva D. A., Vukmirovic R., Davis M. E., Kamm R. D. and Lee R. T. (2004). Endothelial cells promote cardiac myocyte survival and spatial reorganization: implications for cardiac regeneration. *Circulation* 110, 962-968. 10.1161/01.CIR.0000140667.37070.07 15302801 PMC2754572

[DEV143438C31] Orlova V. V., Drabsch Y., Freund C., Petrus-Reurer S., van den Hil F. E., Muenthaisong S., Dijke P. T. and Mummery C. L. (2014). Functionality of endothelial cells and pericytes from human pluripotent stem cells demonstrated in cultured vascular plexus and zebrafish xenografts. *Arterioscler. Thromb. Vasc. Biol.* 34, 177-186. 10.1161/ATVBAHA.113.302598 24158517

[DEV143438C32] Payne R. M. and Strauss A. W. (1994). Expression of the mitochondrial creatine kinase genes. In *Cellular Bioenergetics: Role of Coupled Creatine Kinases* (ed. Saks V. A. and Ventura-Clapier R.), pp. 235-243. Boston, MA: Springer US.

[DEV143438C33] Pointon A., Abi-Gerges N., Cross M. J. and Sidaway J. E. (2013). Phenotypic profiling of structural cardiotoxins in vitro reveals dependency on multiple mechanisms of toxicity. *Toxicol. Sci.* 132, 317-326. 10.1093/toxsci/kft005 23315586

[DEV143438C34] Rasmussen T. L., Shi X., Wallis A., Kweon J., Zirbes K. M., Koyano-Nakagawa N. and Garry D. J. (2012). VEGF/Flk1 signaling cascade transactivates Etv2 gene expression. *PLoS ONE* 7, e50103-e50112. 10.1371/journal.pone.0050103 23185546 PMC3501484

[DEV143438C35] Ravenscroft S. M., Pointon A., Williams A. W., Cross M. J. and Sidaway J. E. (2016). Cardiac non-myocyte cells show enhanced pharmacological function suggestive of contractile maturity in stem cell derived cardiomyocyte microtissues. *Toxicol. Sci.* 152, 99-112. 10.1093/toxsci/kfw069 27125969 PMC4922542

[DEV143438C36] Rolletschek A. (2004). Embryonic stem cell-derived cardiac, neuronal and pancreatic cells as model systems to study toxicological effects. *Toxicol. Lett.* 149, 361-369. 10.1016/j.toxlet.2003.12.064 15093282

[DEV143438C37] Sahara M., Santoro F. and Chien K. R. (2015). Programming and reprogramming a human heart cell. *EMBO J.* 34, 710-738. 10.15252/embj.201490563 25712211 PMC4369310

[DEV143438C111] Sala L., Yu Z., Ward-van Oostwaard D., van Veldhoven J. P., Moretti A., Laugwitz K. L., Mummery C. L., IJzerman A. P. and Bellin M. (2016). A new hERG allosteric modulator rescues genetic and drug-induced long-QT syndrome phenotypes in cardiomyocytes from isogenic pairs of patient induced pluripotent stem cells. *EMBO Mol. Med.* 8, 1065-1081. 10.15252/emmm.201606260 27470144 PMC5009811

[DEV143438C38] Schoors S., De Bock K., Cantelmo A. R., Georgiadou M., Ghesquière B., Cauwenberghs S., Kuchnio A., Wong B. W., Quaegebeur A., Goveia J. et al. (2014). Partial and transient reduction of glycolysis by PFKFB3 blockade reduces pathological angiogenesis. *Cell Metab.*. 19, 37-48. 10.1016/j.cmet.2013.11.008 24332967

[DEV143438C39] Schwach V. and Passier R. (2016). Generation and purification of human stem cell-derived cardiomyocytes. *Differentiation* 91, 126-138. 10.1016/j.diff.2016.01.001 26915912

[DEV143438C40] Skelton R. J. P., Costa M., Anderson D. J., Bruveris F., Finnin B. W., Koutsis K., Arasaratnam D., White A. J., Rafii A., Ng E. S. et al. (2014). SIRPA, VCAM1 and CD34 identify discrete lineages during early human cardiovascular development. *Stem Cell Res.* 13, 172-179. 10.1016/j.scr.2014.04.016 24968096

[DEV143438C41] Stevens K. R., Kreutziger K. L., Dupras S. K., Korte F. S., Regnier M., Muskheli V., Nourse M. B., Bendixen K., Reinecke H. and Murry C. E. (2009). Physiological function and transplantation of scaffold-free and vascularized human cardiac muscle tissue. *Proc. Natl. Acad. Sci. USA* 106, 1-6. 10.1073/iti0109106 PMC2746126 19805339

[DEV143438C42] Tian X., Pu W. T. and Zhou B. (2015). Cellular origin and developmental program of coronary angiogenesis. *Circ. Res.* 116, 515-530. 10.1161/CIRCRESAHA.116.305097 25634974 PMC6914229

[DEV143438C43] Tirziu D., Giordano F. J. and Simons M. (2010). Cell communications in the heart. *Circulation* 122, 928-937. 10.1161/CIRCULATIONAHA.108.847731 20805439 PMC2941440

[DEV143438C44] Tulloch N. L., Muskheli V., Razumova M. V., Korte F. S., Regnier M., Hauch K. D., Pabon L., Reinecke H. and Murry C. E. (2011). Growth of engineered human myocardium with mechanical loading and vascular coculture. *Circ. Res.* 109, 47-59. 10.1161/CIRCRESAHA.110.237206 21597009 PMC3140796

[DEV143438C45] Uosaki H., Fukushima H., Takeuchi A., Matsuoka S., Nakatsuji N., Yamanaka S. and Yamashita J. K. (2011). Efficient and scalable purification of cardiomyocytes from human embryonic and induced pluripotent stem cells by VCAM1 surface expression. *PLoS ONE* 6, e23657-e23659. 10.1371/journal.pone.0023657 21876760 PMC3158088

[DEV143438C46] van den Berg C. W., Elliott D. A., Braam S. R., Mummery C. L. and Davis R. P. (2016). Differentiation of human pluripotent stem cells to cardiomyocytes under defined conditions. *Methods Mol. Biol.* 1353, 163-180. 10.1007/7651_2014_178 25626427

[DEV143438C48] Wang G., McCain M. L., Yang L., He A., Pasqualini F. S., Agarwal A., Yuan H., Jiang D., Zhang D., Zangi L. et al. (2014). Modeling the mitochondrial cardiomyopathy of Barth syndrome with induced pluripotent stem cell and heart-on-chip technologies. *Nat. Med.* 20, 616-623. 10.1038/nm.3545 24813252 PMC4172922

[DEV143438C49] Zeevi-Levin N., Itskovitz-Eldor J. and Binah O. (2012). Cardiomyocytes derived from human pluripotent stem cells for drug screening. *Pharmacol. Ther.* 134, 180-188. 10.1016/j.pharmthera.2012.01.005 22269465

[DEV143438C50] Zhang M., D'Aniello C., Verkerk A. O., Wrobel E., Frank S., Ward-van Oostwaard D., Piccini I., Freund C., Rao J., Seebohm G. et al. (2014). Recessive cardiac phenotypes in induced pluripotent stem cell models of Jervell and Lange-Nielsen syndrome: Disease mechanisms and pharmacological rescue. *Proc. Natl. Acad. Sci. USA* 111, E5383-E5392. 10.1073/pnas.1419553111 25453094 PMC4273331

